# Deltex-1 Activates Mitotic Signaling and Proliferation and Increases the Clonogenic and Invasive Potential of U373 and LN18 *Glioblastoma* Cells and Correlates with Patient Survival

**DOI:** 10.1371/journal.pone.0057793

**Published:** 2013-02-25

**Authors:** Roland M. Huber, Michal Rajski, Balasubramanian Sivasankaran, Gerald Moncayo, Brian A. Hemmings, Adrian Merlo

**Affiliations:** 1 Department of Biomedicine, University of Basel, Basel, Basel-Stadt, Switzerland; 2 Mechanisms of Cancer Program, Friedrich Miescher Institute for Biomedical Research, Basel, Basel-Stadt, Switzerland; 3 Institute of Physiology, University of Zürich, Zürich, Zürich, Switzerland; University of Florida, United States Of America

## Abstract

*Glioblastoma* (GBM) is a highly malignant primary tumor of the central nervous system originating in glial cells. GBM results in more years of life lost than any other cancer type. Low levels of Notch receptor expression correlates with prolonged survival in various high grade gliomas independent of other markers. Different downstream pathways of Notch receptors have been identified. We tested if the Notch/Deltex pathway, which is distinct from the canonical, CSL-mediated pathway, has a role in GBM. We show that the alternative or non-canonical Notch pathway functioning through Deltex1 (DTX1) mediates key features of glioblastoma cell aggressiveness. For example, DTX1 activates the RTK/PI3K/PKB and the MAPK/ERK mitotic pathways and induces anti-apoptotic Mcl-1. The clonogenic and growth potential of established glioma cells correlated with DTX1 levels. Microarray gene expression analysis further identified a DTX1-specific, MAML1-independent transcriptional program - including *microRNA-21*- which is functionally linked to the changes in tumor cell aggressiveness. Over-expression of DTX1 increased cell migration and invasion correlating to ERK activation, miR-21 levels and endogenous Notch levels. In contrast to high and intermediate expressors, patients with low *DTX1* levels have a more favorable prognosis. The alternative Notch pathway via DTX1 appears to be an oncogenic factor in glioblastoma and these findings offer new potential therapeutic targets.

## Introduction

*Glioblastoma* (GBM) is the most common primary tumor of the central nervous system. Despite continuing efforts to improve treatment over the last two decades and advances in microsurgery, radio- and chemotherapy, median survival of patients remained limited at ∼14 months after diagnosis [1]. GBM is a highly aggressive tumor characterized by rapid growth and extensive infiltration of adjacent brain areas. Overall, GBM results in more years of life lost than any other cancer type, cancer-related death is the case in nearly all patients [2].

Notch receptors are evolutionary conserved transmembrane receptors which convey extracellular signals across the cell membrane and trigger signal cascades regulating gene expression. Notch activation has been implicated as a positive determinant of cancer formation in T cell acute lymphoblastic leukemia (T-ALL), primary melanomas, breast cancer and gliomas [3]. Furthermore, Notch signaling was shown to control proliferation and apoptosis in gliomas [4], to promote glioma cell migration and invasion [5] and to promote radio resistance in glioma stem-like cells [6]. Blocking Notch signaling enhanced standard chemo-therapy [7] and depleted the glioma initiating cell pool [8]. Notch ligands provided by endothelial cells induce the self-renewal of cancer stem-like cells in glioblastoma [9]. Previous studies have also shown that loss of Notch2 positively predicts patient survival in subgroups of high grade glial brain tumors [10]. An additional mechanism by which Notch mediates tumor aggressiveness is by the induction of Tenascin-C – an extracellular glycoprotein which correlates with malignancy in glioblastoma and other cancers [11] – by the Notch canonical co-activator RBPJκ [12], [13]. The role of canonical Notch signaling in cancer development, progression and metastasis is intensively studied and evidence is pointing to an oncogenic role of Notch in glioblastoma. However, the role of the non-canonical signaling pathway via Deltex in these mechanisms is still ill defined.

Deltex is a Notch interacting protein which contains a basic region at the N-terminus where it binds to the ankyrin repeats of the intracellular domain of Notch. Deltex has been proposed to regulate Notch activity by antagonizing the interaction between Notch and Suppressor of Hairless [14]. In mammalian cells, *DTX1* has been shown to be a transcriptional target of Notch itself suggesting a positive feedback loop between Notch and DTX1. However, Deltex protein family members contain a RING finger domain at their C-terminus with E3 ubiquitin ligase activity. Deltex has been shown to be part of a three protein complex (containing Notch, Deltex and non-visual β-arrestin) mediating the degradation of the intracellular Notch receptor through a ubiquitination-dependent pathway. Loss-of-function mutations provided *in vivo* evidence for the functional relation of Deltex, Notch and non-visual β-arrestin in Drosophila wing development [15]. Together, Deltex appears to act as a signal repressor or as a mediator of negative feedback for Notch signaling in mammals.

Deltex also exerts its function on Notch independent targets. DTX1 has been shown to exert E3 ubiquitin ligase activity on other protein substrates, such as the mitogen-activated protein kinase signaling component MAP kinase kinase kinase 1 (MEKK1). Targeted MEKK1 degradation by Deltex suppresses the activation of T-cells [16]. In mice, three new ligands to the Notch receptor family have been identified which signal specifically through the DTX1 pathway [17]-[19] independently of RBPJκ and MAML1 and one of these ligands (DNER) has been implicated in non-canonical regulation of glioma inducing cells [20]. However, the genes involved in this pathway remain ill-defined [21], [22]. In summary, Deltex constitutes a distinct, cell context-dependent Notch signaling pathway.

Regarding the cellular origin of gliomagenesis, several findings suggest progenitor or adult stem cells as possible founder cells of intracranial neoplasms [23]. Most interestingly, Deltex has been shown to block the transcription factor MASH1 in neural progenitor cells by binding to p300 and thereby blocking differentiation of these cells. This differentiation block was shown to be independent of canonical Notch signaling via RBPJκ [24]. Furthermore, a Deltex mediated block of neural differentiation has been shown in microchaetae sensory precursors in Drosophila indicating a conserved role for Deltex as a regulator of differentiation in neuroglial tissues [25].

In this study we provide evidence that DTX1 has an oncogenic role in high grade glioma cell lines. We provide molecular insight in how a modulation of DTX1 levels changes the signaling networks in cancer cells and relate these findings to changes in the proliferative, migratory and clonogenic potential. We identify a set of genes specifically controlled by this non-canonical Notch signaling pathway and its impact on tumor phenotype and aggressiveness. Finally, low DTX1 expression levels correlate with longer survival in GBM and breast cancer patients.

## Materials and Methods

### Cell culture and cell lines

Glioma cell lines (LN18, U373) with defined genetic status for TP53, p16/p14 and PTEN [26] were cultured in Eagle’s medium supplemented with 25 mM glucose, L-glutamine, standard antibiotics, and 10% fetal calf serum. All cells were maintained at 37°C in 5% CO_2_ in a humified chamber (standard conditions). If not stated otherwise in the figure legends, cells were seeded at 5’000-10’000 cells/cm^2^ in 94 mm culture dishes (Sarsted, Nümbrecht, Germany). For cell counting, the cells were cultured and treated as stated in the figure legends and counted using a ‘Neubauer’-chamber (hemacytometer).

### Plasmids, siRNA and transfections

The lentiviral vectors pLKO.1-puro-scrambled-shRNA (Addgene, Cambridge, MA, USA) and pLKO.1-puro-shRNA (Sigma, sh1938: CCGGC CACTG CTATC TACCC AACAA CTCGA GTTGT TGGGT AGATA GCAGT GGTTT TT) targeting DTX1 were transfected into HEK293 cells together with plasmids encoding the packaging (pCMV_dr8_91) and envelope proteins (pMD2-VSV-G) using CaCl_2_ precipitation. The concentration of infectious particles in the supernatant was titrated using HeLa cells. Glioma cells were transduced with infectious viral particles. Stably transfected clones were selected with 2 µg/ml puromycin. DTX1 over-expression was obtained with pcDNA3-IRES-EGFP as control and pcDNA3-DTX1-myc-IRES-EGFP (kind gift form Prof. Kimie Ohta, Keio University, Japan) using CaCl_2_ precipitation for 8 hours. Stably transfected clones were selected with 100 µg/ml geniticin (Gibco, Invitrogen, San Diego CA, USA). For siRNA experiments we used pools of at least 6 siRNAs targeting the protein of interest according to the manufacturer’s instructions (“on-target plus smart pool siRNA”, Dharmacon, Waltham, MA, USA).

### RT-PCR and qPCR

RNA was extracted using TRI-Reagent (Sigma, St.Louis MO, USA), phenol/chloroform extraction and were purified with RNeasy spin column kit (Qiagen, Venlo, The Netherlands). cDNA synthesis was performed using the ‘Thermo Script RT-PCR System’ and random primer hexameres (Invitrogen, San Diego CA, USA). DTX1 primers: fwd (5′- GGGCT GATGC CTGTG AATG-3′), rev (5′- CCTGG CGAAA CTGGT GC-3′). RNA and cDNA amounts were measured on a NanoDrop ND-1000 Spectrophotometer and equalized prior to synthesis reactions. Semi-quantitative PCR was performed as described elsewhere [27]. qPCR for p300 was performed as described elsewhere [28]. microRNAs were isolated using miRNeasy Mini Kit (Qiagen) according to manufacturer’s instructions. Taqman MicroRNA Reverse Transcription Kit and Megaplex RT Primers human pool A v 2.1 (Applied Biosystems) were used for cDNA synthesis of microRNAs and Taqman Pri-miRNA Assay for the measurement of miR-21 levels according to manufacturer’s instructions (Applied Biosystems).

### Cell proliferation analysis

Cell proliferation was analyzed using the ‘Amersham Cell Proliferation Biotrak ELISA, version 2’ system (GE Helathcare, UK) according to manufacturer’s instructions. In short, 5’000 cells were seeded in the well of a 96-well plate and grown for two days, labeled with BrdU for 3–4h, fixed and labeled with a peroxidase-labeled anti-BrdU antibody. After coloring reaction the optical density was measured with a ‘SpectraMAX 250’ plate reader and analyzed with accompanying ‘Soft Max Pro’ software (Molecular Devices, MDS Analytical Technologies, Toronto, Canada). For cell counting, equal amounts of cells were seeded in triplicates and grown under standard conditions for three and five days. Cells were then harvested and each biological replicate was counted ≥8 times using a ‘Neubauer’-chamber (hemacytometer).

### Colony formation, sphere and soft agar assay

In colony formation experiments for each cell line 1’000 cells were plated in triplicates into 94-mm Petri dishes containing 10 ml of standard culture medium. Cells were then fixed with 4% formaldehyde in 1x PBS and stained with crystal violet. For sphere formation, cells were seeded in Neurobasal medium (Invitrogen) supplemented with basic FGF (20 ng/ml, Invitrogen), EGF (20 ng/ml, R&D Systems), B27 supplement (1x) and N_2_ supplement (0.5x) (Invitrogen) and grown under standard conditions (see above) for different times: shRNA-scr and shRNA-DTX1 for 24d, EGFP and DTX1-myc cells for 15d. In soft agar experiments for each cell line 1’000 cells were seeded in 0.3% agar (Nobel Agar, Becton Dickinson, USA) in DMEM supplemented with 10% FCS without phenol red (Gibco, Invitrogen, San Diego CA, USA) and grown for 15 days under standard conditions. All spheres were documented with an ‘Olympus IX50’ microscope using the ‘Color View Soft Image System’ controlled by ‘cellP’ software (Olympus, Tokio, Japan).

### Transwell migration and scratch test assay

Transwell migration assays were performed using modified Boyden chamber units with polycarbonate filters of 8 µm porosity (Costar, Vitaris, Switzerland). The lower side of the filter was coated with 25 µg/ml collagen 1 (Sigma, St.Louis, USA) for 2h at 37°C. The bottom chamber was filled with DMEM containing 10% FCS. Cells (2×10^4^ per well in serum-free DMEM) were plated in the upper chamber in 100 µl medium and incubated for 24h in standard conditions (see above). After removal of the remaining cells from the upper surface of the filter insert, migrated cells at the bottom of the filter were fixed with 3.7% formaldehyde in PBS and stained with 0.1% crystal violet. For every individual filter, the cells in 9 fields of view were counted. Every experiment was conducted in triplicates.

For scratch test analysis, cells were grown to ∼90% confluency under standard conditions. A wound was inflicted by scratching a 200 µl pipette tip (Starlab, Milton Keynes, UK) over the surface of the culture flask. The wounds were documented as described above for the soft agar immediately after scratching, after 12, 24 and 48 hours. Quantification of wound closing was performed with ImageJ software according to manufacturers’ instructions.

### Western blot analysis and antibodies

Cells were grown to 80–90% confluency, washed twice with 1x PBS, lysed in buffer containing 2% sodium dodecyl sulfate (SDS), 50 mM Tris pH 6.8, 0.1 M dithiothreitol (DTT), boiled at 95°C for 5 min and used either immediately or frozen at -20°C. Protein lysates were resolved on denaturing 8–12% SDS-polyacrylamide gels and transferred to nitrocellulose membranes (iBlot Gel transfer stacks, Invitrogen). The following primary antibodies were used: anti-Actin (Sigma-Aldrich, St. Louis, USA), anti-phospho-Akt/PKB and anti-total-Akt/PKB (Ser-473) (Millipore), anti-Akt2/PKBβ (Cell Signaling), anti-DTX1 (ABBiotech), anti-EGFR (Santa Cruz Biotechnology), anti-phospho-Erk and anti-total-Erk (Santa Cruz Biotechnology), anti-Mcl-1 (Santa Cruz Biotechnology), anti-Myc Tag (Millipore) and anti-Snail1 (Abcam). Decorated proteins were revealed using horseradish peroxidase-conjugated anti-mouse, anti-rabbit, anti-rat (New England Biolabs) or anti-goat (Pierce) secondary antibodies and visualized by the chemoluminescence detection system SuperSignal West Pico (Thermo Scientific). Densitometry of western blots was performed using ImageJ software according to manufacturers’ instructions; values of representative blots are shown in the figures. Radio-densitometry was performed using ImageJ software according to manufacturer’s instructions.

### Microarray analysis of patient biopsies and cell lines

Microarray gene expression analysis of patient biopsies has been reported earlier [29]. This data set was reanalyzed in respect of *DTX1* expression. For cell lines, total RNA was extracted from U373-EGFP, U373-DTX1-myc and U373-MAML1-dn cells in triplicates and amplified once. Samples were hybridized to Affymetrix chips HuGene 1.0 ST v1. The data analysis and gene filtering was performed using R/Bioconductor [30]. Signal condensation was performed using only the RMA from the Bioconductor Affy package. Differentially expressed genes were indentified using the empirical Bayes method (F-test) implemented in the LIMMA package and adjusted with the False Discovery Rate (FDR) method [31]. We selected those probe sets with a log2 average contrast signal of at least 4, an adjusted p value <0.01 and an absolute linear fold change of >2. The gene expression data has been deposited at the Gene Expression Omnibus Databank (accession ID: GSE22772) according to MIAME standards. Hierarchical clustering and visualization was performed in R software. The scripts are available upon request. Assessment of the uncertainty in hierarchical cluster analysis was performed with “pvclust” package as described elsewhere [32] based on all annotated genes or 191 annotated genes specifically alerted by DTX1 as described in the text.

### Patient survival data mining

Patient survival plots for various genes of interest (eg. *DTX1, PKBβ, EGR1*, etc.) can be accessed here: https://caintegrator.nci.nih.gov/rembrandt/ .

## Results

### Deltex 1 is expressed in primary gliomas and glioma-derived cell lines

**Figure 1:**
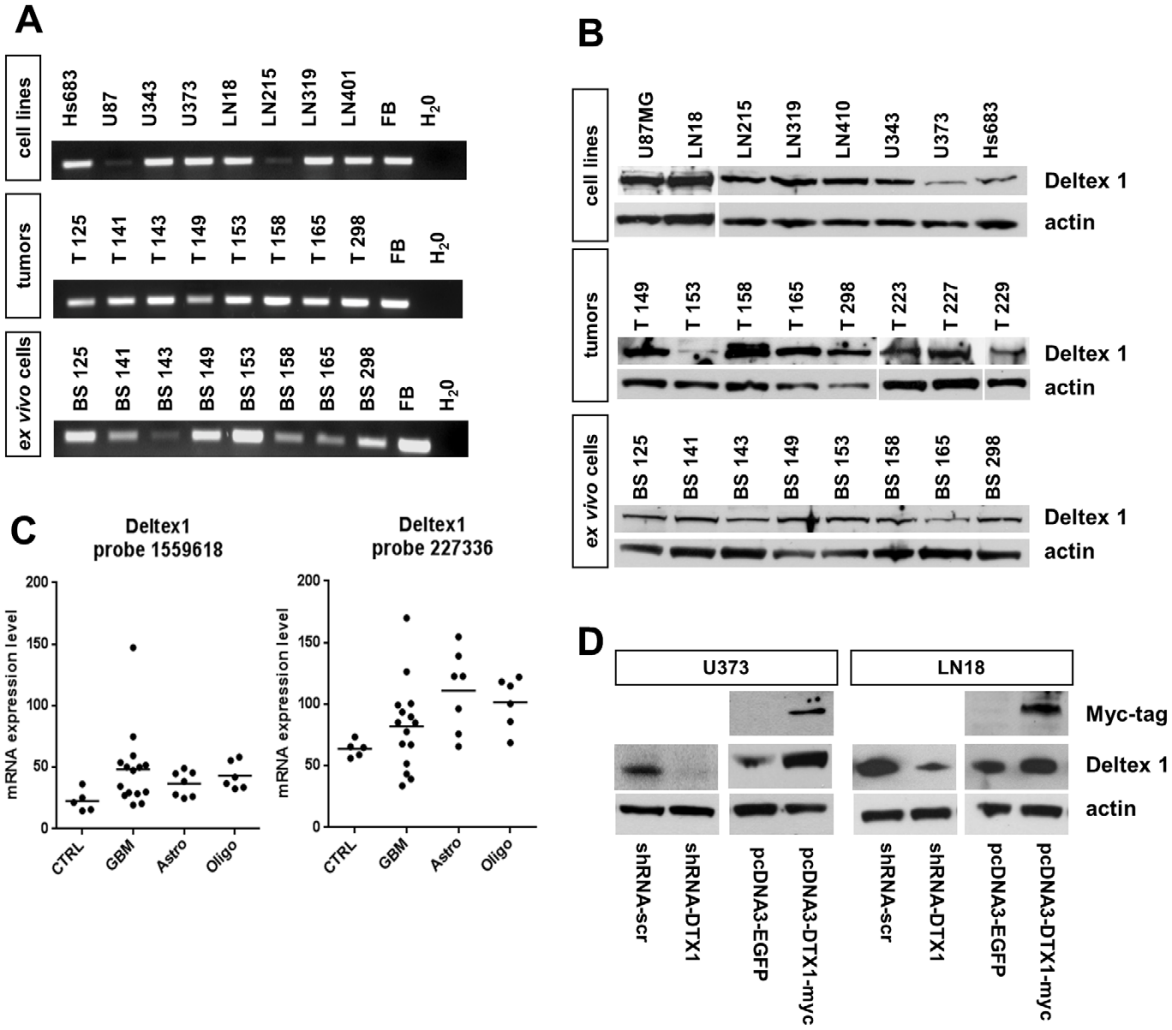
DTX1 is expressed in glioblastomas, *ex vivo* cells and established glioma derived cell lines. (A) Semi-quantitative RT-PCR probing for exon 1 of DTX1 in established glioma derived cell lines, glioma tumor biopsies and *ex vivo* cell lines. *Ex vivo* cell lines were derived from tumor biopsies as indicated by the numbering and were maintained as low passage cultures. Fetal brain (FB) was used as positive control. (B) Western Blot analysis of glioma derived cell lines, glioma tumor biopsies and *ex vivo* cell lines probing for DTX1 and β-actin. (C) Microarray gene expression analysis of tumors and control tissue. Two non-diseased normal brain samples and three normal human astrocyte cultures were used as controls (ctrl), 15 GBMs, seven astrocytomas and six oligodendrogliomas were analyzed. Dots represent individual specimens, average expression values are shown as lines. Results for two independent probes on the chip detecting DTX1 mRNA are shown. (D) Western blot analysis of transfected cell lines U373 and LN18 probing for DTX1, Myc-tag and β-actin demonstrating DTX1 over-expression and down regulation as indicated according to the genotype.

We analyzed *Deltex1* (*DTX1)* expression both at the transcript and the protein level in tumor biopsies and glioma-derived cell lines to confirm its presence in gliomas. First, we performed semi-quantitative RT-PCR with cDNA derived from established glioma cell lines, tumor biopsies directly derived from the operating room and with low passage *ex vivo* glioma cells generated in our laboratory. All established cell lines showed *DTX1* expression at varying levels. All tumor biopsies and all *ex vivo* cells showed robust *DTX1* expression at the transcript level (Figure 1, A). We performed RT-PCR based transcript mapping, confirming in all probes full length transcripts by the presence of exon 1, 2, 3–5, 6–8, and 9 as well as of the 3’UTR of the mRNA with individual primer sets (data not shown). We found expression of DTX1 protein at varying levels in all glioma cell lines, tumor biopsies and *ex vivo* cell lines analyzed by immunoblotting (Figure 1, B), confirming the transcript analysis.

To further validate our results, total RNA was isolated from two normal human brain controls, three normal human astrocyte cultures, 15 *glioblastoma*, seven astrocytomas (grade II–III) and six oligodendrogliomas, and processed for gene expression analysis by microarrays [29]. Two independent probes detecting *DTX1* mRNA (1559618_at and 227336_at, Affymetrix) confirmed *DTX1* expression in all glioma samples analyzed. Although elevated average levels of *DTX1* expression were found in glioma samples, this difference was not significant (p = 0.094) (Figure 1, C). Altogether, we found DTX1 to be expressed in glioma biopsies and cell lines both at the transcript and protein level.

To further investigate the role of DTX1, we generated cell lines over-expressing DTX1 (pcDNA3-DTX1-myc) and glioma cells with reduced levels of DTX1 through shRNA interference (pLKO.1-shRNA-DTX1) (Figure 1, D). We used two established cell lines which differ in their endogenous Notch status: U373 expresses high endogenous levels of Notch1 and Notch2, whereas LN18 shows low expression levels for these two receptors [12]. This allowed us to relate DTX1 modification effects to the underlying activity of the Notch pathway.

### Deltex 1 regulates signaling pathways of cell growth and survival

Unlimited replicative potential of tumor cells is a hallmark of aggressive cancers. Notch acts as a switch-like signaling mechanism controlling the transition from proliferation to differentiation and vice versa depending on the cellular context. We therefore analyzed several signaling pathways in relation to the DTX1 status of the cell which are known to control proliferation and are often altered in GBM [33].

**Figure 2:**
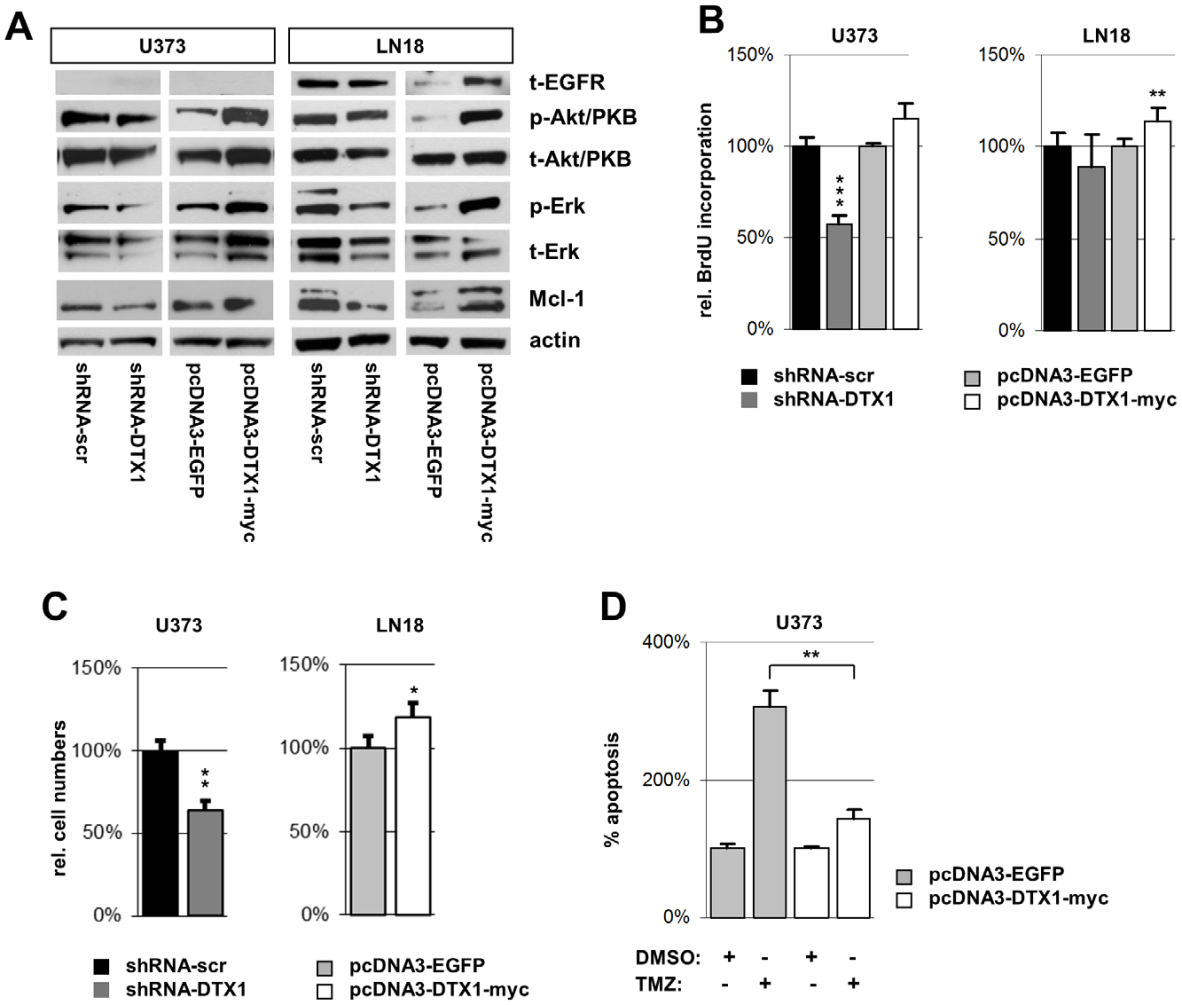
Proliferation and mitotic signaling pathways are modified by DTX1 in established glioma cell lines. (A) Western blot analysis of signaling cascade proteins. Blots for total EGFR (t-EGFR), phosphorylated Akt/PKB (p-Akt/PKB), total Akt/PKB (t-Akt/PKB), phosphorylated Erk (p-Erk), total Erk (t-Erk), Mcl-1 and β-actin (actin) are shown. (B) Proliferation analysis by BrdU incorporation assay. Relative average values of 5 individual experiments are shown per genotype. (C) Proliferation analysis by cell counting. Equal numbers of cells were seeded, grown for 3d under standard conditions and counted afterwards. (D) Apoptosis in U373 cells after treatment with 100 µM temozolomide (TMZ) or DMSO control. Relative values of sub-G_1_ cells measured by PI staining are shown normalized to control cells treated with vector control. Averages of at least three independent experiments are shown. Values are normalized to controls. Error bars: ±SEM. * p<0.05, ** p<0.01, *** p<0.001.

In gliomas, the PI3K/PKB/mTOR and the MAPK/ERK pathways are hyper-activated by specific mutations [34]. We analyzed the effect of DTX1 on the expression level of EGF receptor (EGFR), a receptor tyrosine kinase frequently over-expressed in gliomas. In the EGFR-expressing glioma cell line LN18, DTX1 over-expression increased EGFR protein levels. However, shRNA-DTX1 only marginally reduced EGFR levels (Figure 2, A). In the U373 cell line that does not express EGFR, we could not induce *de novo* expression of EGFR by DTX1 (Figure 2, A). The levels of p-EGFR were not significantly changed (data not shown). In both LN18-DTX1-myc and U373-DTX1-myc we observed an increase of the phospho-Akt/PKB (p-Akt/PKB) levels. In contrast, we detected a slight reduction of p-Akt/PKB levels in the LN18-shRNA-DTX1 and U373-shRNA-DTX1 cells. In both glioma cell lines analyzed, down-regulation of DTX1 reduced the levels of phosphorylated Erk (p-Erk). On the other hand, DTX1 over-expression induced p-Erk (Figure 2, A). The changes observed for p-Akt/PKB and p-Erk levels were paralleled by changes in the total amounts of the proteins. The anti-apoptotic protein Mcl-1 was reduced by shRNA-mediated DTX1 down-regulation and induced in DTX1-myc over-expressing cells in both cell lines (Figure 2, A).

To determine how these changes in MAPK and PI3K/PKB signaling affect proliferation, we performed BrdU incorporation assays to detect alterations in tumor cell proliferation in relation to DTX1 levels. U373-DTX1-myc cells did not show any significant changes whereas U373-shRNA-DTX1 cells displayed a significant reduction by 35% (p<0.001, ***) (Figure 2, B). In LN18 cells, over-expression of DTX1 increased proliferation by 14% (p<0.01, **). However, down-regulation did not significantly change the proliferative behavior of LN18 cells (Figure 2, B). In accordance with the results obtained in the BrdU incorporation assay, we observed a reduction by 35% (p<0.01, **) when analyzing cell numbers after 3 days of unchallenged growth in U373-shRNA-DTX1 cells (Figure 2, C) (similar results were seen after 5 days, data not shown). LN18-DTX1-myc cells showed a 19% increase in total cells (p<0.05, *) (Figure 2, C). Background apoptosis was not changed in any of the cell lines compared to controls as analyzed by sub-G_1_ cell content (data not shown). When challenged with 100 µM temozolomide (TMZ) relative apoptosis in U373-EGFP increased to 305% of control but only to 143% in U373-DTX1-myc cells (p < 0.01, **) (Figure 2, D). There was no significant difference between U373-shRNA-scr and U373-shRNA-DTX1 cells regarding TMZ sensitivity (data not shown).

These results show that two major survival and growth promoting signaling pathways and the anti-apoptotic protein Mcl-1 are positively regulated by DTX1 in established glioma derived cell lines. Proliferation and drug resistance correlated with these findings and the effects also correlated with the endogenous Notch status of the underlying cell line. A direct link between DTX1 and the MAPK and PI3K/PKB pathways has so far not been described; we therefore aimed to identify molecular mediators involved.

### Deltex 1 controls gene expression in glioma derived cells and regulates genes independently of the canonical Notch signaling cascade

We compared U373-EGFP control cells to U373-DTX1 cells by microarray gene expression profiling experiments that allow the identification of genes controlled by non-canonical Notch signaling. Given the tight interaction of DTX1 with Notch and the negative feedback loop on the canonical pathway (Figure S1, A), we aimed to exclude genes which are altered due to this effect of DTX1 on the canonical pathway. Therefore, we compared the first dataset to the gene expression profiles of U373 cells expressing a dominant negative form of MAML1 (MAML1-dn) mimicking the negative feedback of DTX1 on canonical Notch signaling (Figure S1, B).

We identified 763 annotated genes to be differentially expressed in U373-DTX1 cells (665 up, 98 down) and 991 in U373-MAML1-dn (872 up, 119 down) when compared to EGFP control cells (Table S1; Figure S1, C). Several genes known to be regulated by canonical Notch signaling and which have previously been implicated in cancer biology (eg. *Rb, Twist, PIK3C, MAPK2, HER2 or HIF-1α*) [35] showed comparable changes in their expression profiles in both DTX1 and MAML1-dn cells. 572 genes were commonly affected in both cell lines compared to U373-EGFP control cells, 419 uniquely in U373-MAML1-dn and 191 uniquely in U373-DTX1 cells (Figure S1, D). Out of the 191 annotated transcripts unique to U373-DTX1 cells (Table S3), 121 were significantly up-regulated and 70 were significantly down regulated more than 2 fold compared to EGFP cells (Table S1).

Unsupervised clustering based on overall gene expression profiles pointed towards a high similarity between MAML1-dn and DTX1 transcriptomes. This confirms the negative feedback role for DTX1 on canonical Notch signaling resulting in correlated effects (p<0.05) on overall gene expression (Figure S1, A, C and S2, A). Unsupervised clustering based on the annotated 191 genes specifically altered in U373-DTX1 cells showed high specificity for this DTX1 gene set compared to both EGFP and MAML1-dn expressing cells (p<0.05; Figure S2, B). To confirm the validity of these two clusters, we assessed the uncertainty in hierarchical cluster analysis. For each cluster, *p*-values were calculated via multiscale bootstrap resampling [32]. These results confirmed that both clusters (Figure S2, A, B) are strongly supported by data (p < 0.05).

Next we analyzed the functional groups of DTX1-specific genes according to their gene ontology (GO). Most genes belong to metabolic (23 genes) or signal transduction (22 genes) networks. Among others, the GO categories cell cycle control, cell motion, extracellular matrix and apoptosis also contained 30 genes controlled by DTX1 suggesting a direct mechanistic link between the phenotypes observed and gene expression (Table S2). We next performed an extensive literature search for all 191 genes specific for DTX1 with respect to their functional role in cancer and GBM in particular. Some 20 genes with described functions in cancer specifically controlled by DTX1 are shown in Table 1. Interestingly, we observed an induction of *miR-21*, a microRNA which has previously been shown to target a network of tumor suppressive pathways in GBM cells including the p53, the TGF-β and the mitochondrial apoptosis tumor-suppressive pathways [36]. Taken together, these results identify a set of genes specifically controlled by non-canonical Notch signaling through DTX1 including known oncogenes and oncomirs and indicate a negative feedback role of DTX1 on canonical Notch signaling.

**Table 1:** Differentially expressed genes upon DTX1 signaling modification.

Accession ID	Gene Symbol	Protein Name	fold change
examples of genes up regultated >2-fold and related to cancer			
NM_004416	DTX1	deltex homolog 1 1)	47.2
NM_005264	GFRA1	GDNF family receptor alpha 1	3
AY699265	MIRN21	microRNA 21	2.85
NM_001018159	NAE1	NEDD8 activating enzyme E1 subunit 1	2.54
NM_018131	CEP55	centrosomal protein 55kDa	2.49
NM_012096	APPL1	adaptor protein, phosphotyrosine interaction, PH domain and leucine zipper containing 1	2.37
NM_003238	TGFB2	transforming growth factor, beta 2	2.16
NM_001025366	VEGFA	vascular endothelial growth factor A	2.14
NM_005921	MAP3K1	mitogen-activated protein kinase kinase kinase 1	2.05
ENST00000321331	HIGD1A	HIG1 domain family, member 1A	2.04
NM_175866	UHMK1	U2AF homology motif (UHM) kinase 1	2.02
examples of genes down regulated >2-fold and related to cancer			
NM_001040058	SPP1	secreted phosphoprotein 1	0.27
NM_003811	TNFSF9	tumor necrosis factor (ligand) superfamily, member 9	0.28
NM_002346	LY6E	lymphocyte antigen 6 complex, locus E	0.3
NM_001946	DUSP6	dual specificity phosphatase 6	0.41
NM_020645	NRIP3	nuclear receptor interacting protein 3	0.43
NM_016086	STYXL1	serine/threonine/tyrosine interacting-like 1	0.45
NM_002231	CD82	CD82 molecule	0.46
NM_030964	SPRY4	sprouty homolog 4	0.47
NM_001964	EGR1	early growth response 1	0.48

DTX1 over-expressing cell line.

### miR-21 expression is controlled by DTX1 and is p300 dependent

**Figure 3:**
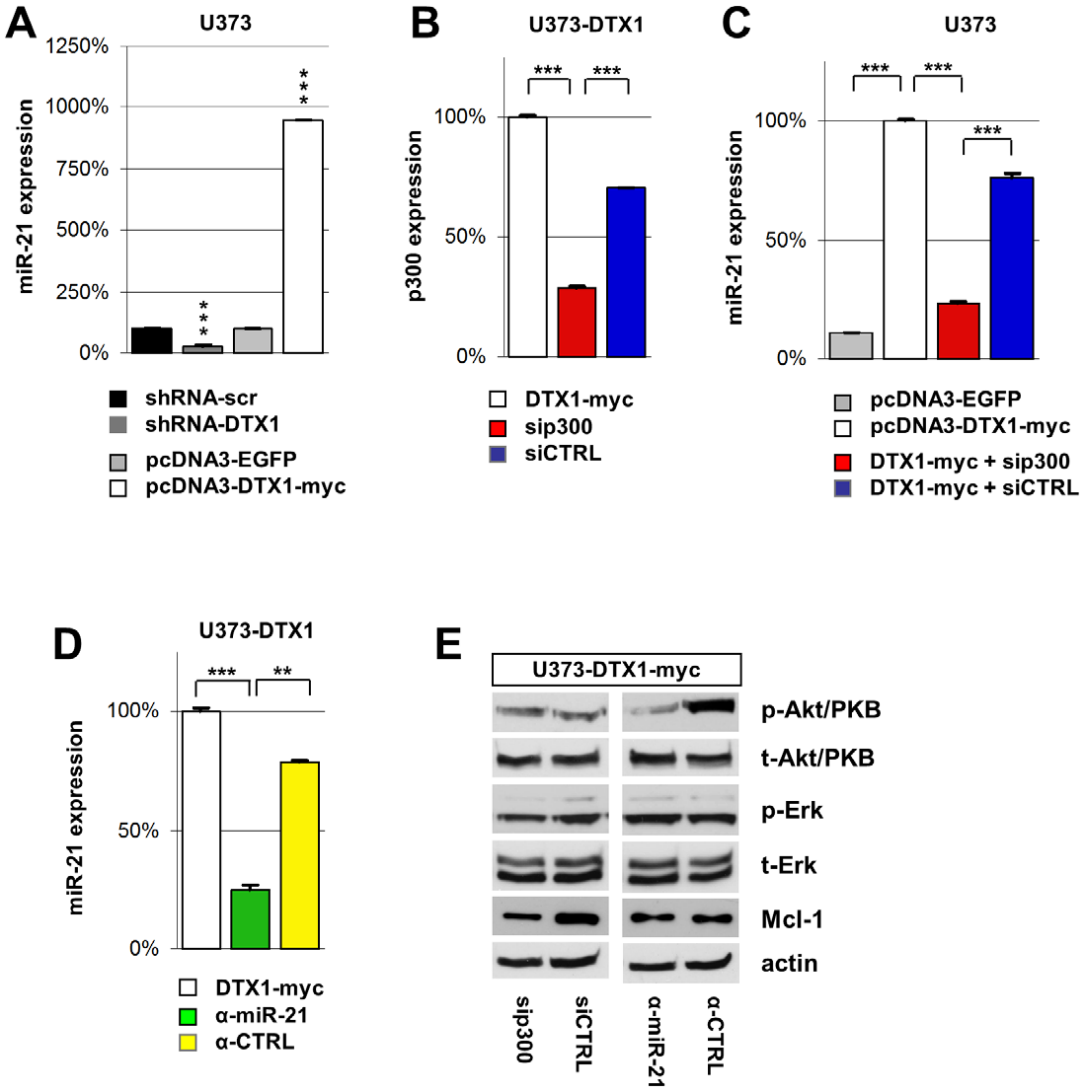
DTX1 controls miR-21 expression in a p300 dependent manner. (A) Real time quantitative PCR analysis of miR-21 expression in U373 cells with modified DTX1 levels. (B) p300 and (C) miR-21 expression levels in U373-DTX1-myc cells transfected with siRNA targeting p300 (sip300, red) or control siRNA (siCTRL, blue) analyzed by qPCR normalized to U373-DTX1-myc. (D) miR-21 expression in U373-DTX1-myc cells treated with miR-21 inhibitor (α-miR-21, green) or an inhibitor control (α-CTRL, yellow). Average relative expression values of at least four independent experiments are shown. Error bars: ±SEM. * p<0.05, ** p<0.01, *** p<0.001. (E) Western blot analysis of signaling cascades in U373-DTX1-myc cells transfected with sip300 or siCTRL. Blots were probed for phosphorylated and total Akt/PKB, phosphorylated and total Erk, Mcl-1 and actin.

To evaluate the results obtained by microarray analysis, we performed quantitative PCR measurements of miR-21 levels. We found a reduction of miR-21 in U373-shRNA-DTX1 to only 30% of the control levels (p< 0.001, ***) whereas over-expression of DTX1 resulted in an increase of miR-21 to 951% of the control (p < 0.001, ***) (Figure 3, A) confirming our microarray data.

Previous studies have identified the histone acetyl-transferase p300 as a binding partner of DTX1 which in turn modulates p300’s co-activator activity in various contexts [24]. Therefore, we analyzed the effect of p300 knock-down on the DTX1 induced miR-21 expression. We transiently transfected U373-DTX1-myc cells with a siRNA-pool targeting p300 (sip300) or scrambled control siRNAs (siCTRL) alongside a GFP vector. After 2 days, >80% of the cells showed GFP expression indicating high transfection efficiency (data not shown). p300 levels were analyzed by qPCR showing effective targeting of p300 (Figure 3, B). DTX1-myc cells transfected with sip300 showed reduced miR-21 expression remaining with 23% of DTX1-myc control or 31% of siCTRL levels (p < 0.001, ***) (Figure 3, C). Hence, miR-21 activation by DTX1 is partially depending on the DTX1-p300 complex. Transfection of U373-DTX1-myc cells with miR-21 inhibitor (α-miR-21) reduced miR-21 levels to 25% compared to DTX1-myc cells (Figure 3, D) (p < 0.001, ***) and to 32% compared to cells treated with an inhibitor control (α-CTRL) (Figure 3, D) (p < 0.01, **).

Inhibition of miR-21 resulted in a reduction of p-Akt/PKB levels in DTX1-myc cells whereas the down-regulation of p300 did neither affect phosphorylation nor total Akt/PKB protein levels (Figure 3, E). Similarly, Erk phosphorylation and total amounts were not affected upon inhibition of miR-21 (Figure 3, E). Mcl-1 expression was reduced in U373-DTX1-myc cells treated with sip300 but was unchanged in α-miR-21 treated cells (Figure 3, E). Taken together, these data demonstrate that miR-21 expression is controlled by DTX1. The induction of miR-21 and Mcl-1 is p300-dependent while activation of Akt/PKB depends on miR-21.

### The clonogenic potential of established glioma cells is regulated by Deltex 1

The ability to initiate clonogenic growth – a determinant of tumorigenicity – is reflected by the potential to form colonies *in vitro* when seeded at very low density, minimizing cell-cell contact, when grown in an anchorage independent manner in low density soft agar or as floating spheres in neurobasal medium [37]. We therefore analyzed the clonogenic potential of our modified glioma cell lines in these experimental settings.

**Figure 4:**
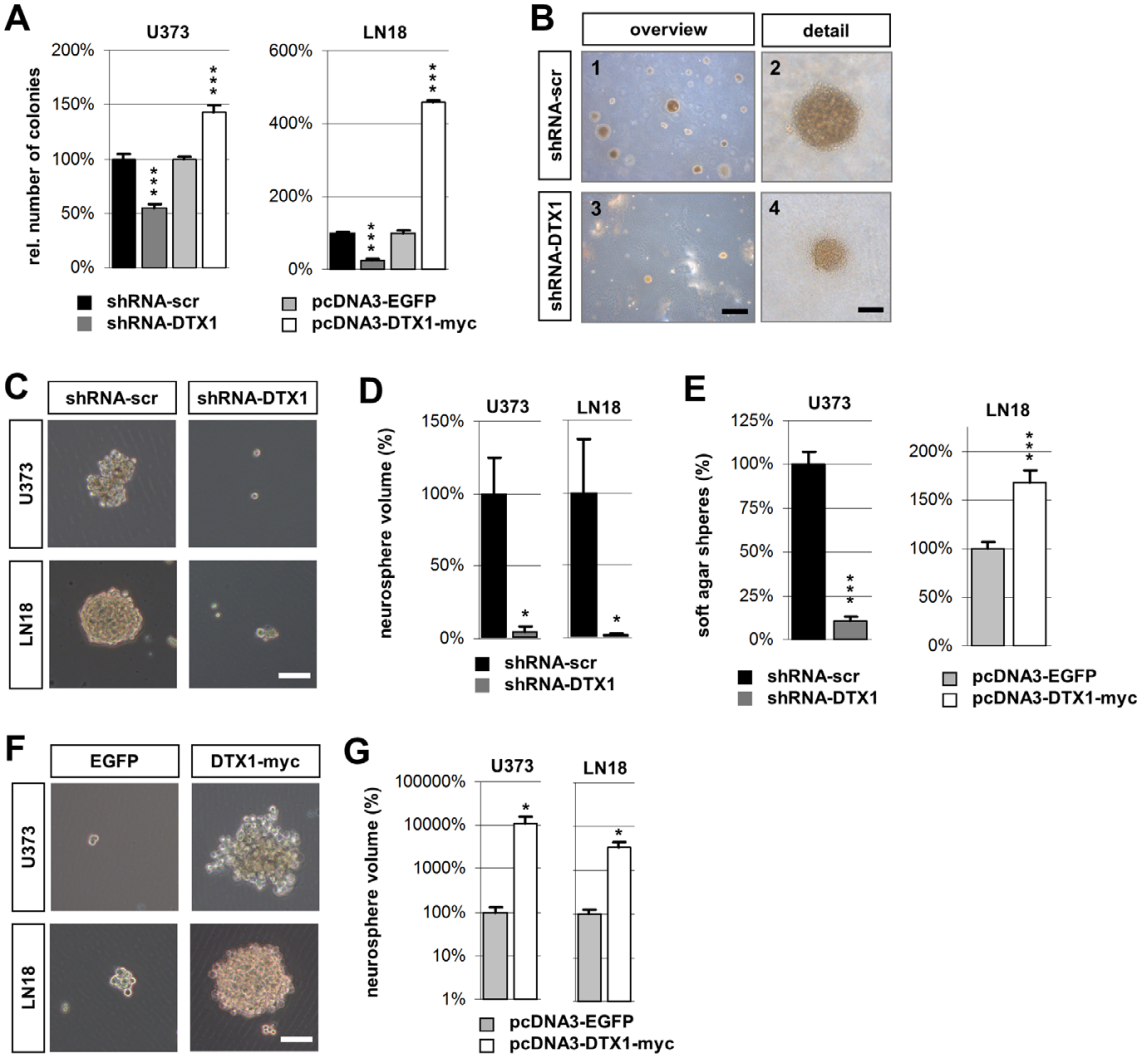
DTX1 regulates the clonogenic capacity of U373 and LN18 glioma cells. (A) Quantification of colonies formed in low density seeding assay after 21d. All colonies were included irrespective of size. Values were normalized to the control cell line. (B) Soft agar colony formation assay shown as light microscopic overview images (1, 3) or detailed close up images (2, 4) of a representative individual colony showing surface independent growth in 0.3% agar. Scale bars: overview 400 µm; detailed view 80 µm. (C) Floating cancer spheres of control (left) or DTX1 down regulated (right) cells grown in NBE medium for 24 days shown as light microscopic images. (D) Relative average volume of neurospheres shown in (C). (E) Quantitative analysis of the number of colonies formed after 15d of incubation in soft agar shown in (B). (F) Floating cancer spheres of control (left) or DTX1 over expressing cells (right) grown in NBE medium for 5 days shown as light microscopic images. (G) Relative average volume of neurospheres shown in (F). Scale bars (C, F): 150 µm. Average values are shown. Error bars: ±SEM. *p<0.05, *** p<0.001.

U373 and LN18 cells transfected either with EGFP, DTX1-myc, shRNA-DTX1 or shRNA-scr were seeded at low density, grown for 3 weeks, fixed and stained with Cresyl violet. Over-expression of DTX1 significantly increased the number of colonies compared to EGFP controls. We observed 43% more colonies and 358% more colonies in U373 and LN18, respectively (p<0.001, ***) (Figure 4, A; Figure S3, A). U373-shRNA-DTX1 cells showed a significant reduction in colony formation by 42% (p<0.001, ***). Down regulation of DTX1 in LN18 cells resulted in a reduction by 73% (p<0.001, ***) (Figure 4, A; Figure S3, A). The overall area covered by individual colonies appeared smaller in DTX1 over-expressing LN18 cells. However, cell density was massively increased in these colonies. Furthermore, LN18-DTX1 cells showed a pronounced phenotype of aggregation and grew in multiple layers, which was not observed in control cells (Figure S3, B).

Anchorage-independent cell growth in soft agar was also dependent on DTX1 status. While the shRNA-scr control cells readily formed spheres with considerable density, the shRNA-DTX1 cells only formed very few colonies. On average, these colonies were also smaller than the control spheres (Figure 4, B). In total, a reduction of around 90% of growing spheres was observed in the case of DTX1 down regulation in U373 (p < 0.001, ***) (Figure 4, E). Over-expression of DTX1 in LN18 cells resulted in a 68% increase in colony number (p < 0.001, ***) (Figure 4, E). Over-expression of DTX1 in U373 or shRNA-DTX1 in LN18 did not significantly change sphere formation. miR-21 inhibition did not change the clonogenic potential of glioma cells when plated at low density (Figure S3, C, D).

Cells were seeded in serum-free neurobasal medium to check for differences in floating cancer sphere formation as an additional measure for the oncogenic effect of DTX1. Down-regulation of DTX1 reduced both colony numbers and size in both cell lines (Figure 4, C, D). U373-DTX1-myc cells formed more and larger spheres when compared to EGFP control cells. Likewise, LN18-DTX1-myc cells formed larger spheres while control cells did only form few and small spheres (Figure 4, F, G). Taken together, these results show a significantly reduced aggressiveness of established glioma cells with down regulated DTX1 as shown by the reduced ability to give rise to cell colonies or spheres in all assays performed, an effect we found to be insensitive to miR-21 inhibition.

### Deltex 1 controls cell migration and invasiveness in established glioma cells

GBM are highly invasive neoplasms. We have previously shown that Notch signaling induces Tenascin C (TnC), a marker for tumor malignancy which stimulates glioma cell migration and invasion through the canonical Notch pathway. This induction was RBPJκ-dependent and could be blocked by using a dominant negative form of the Notch canonical co-activator MAML1 (Sivasankaran et al., 2009). However, it remained unanswered if the non-canonical Notch pathway also has a regulatory role in cell migration and invasiveness.

**Figure 5:**
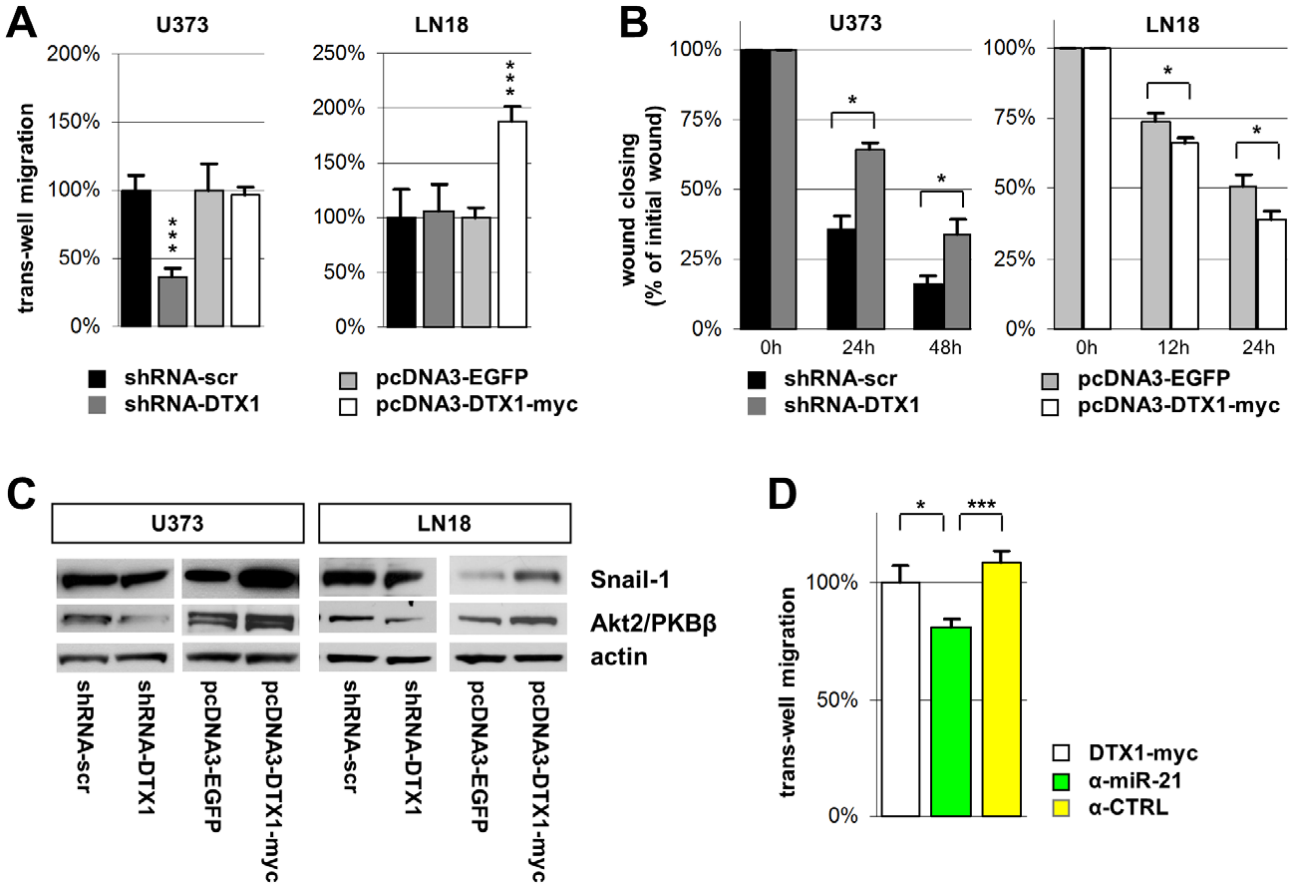
Migration and invasion potential of glioma cells is regulated by DTX1. (A) Boyden chamber trans-well migration and invasion assay with U373 and LN18 glioma cells on collagen coated membranes with 8 µm porosity. Counts were performed after 24h. (B) Scratch test wound healing assay. A scratch wound was inflicted and immediately imaged (time 0h). Follow up images were taken after 12, 24 and 48 hours. Wound closing was assessed using standard imaging software. (C) Western blot analysis of known pro-migratory factors in glioblastoma probing for Snail-1, Akt2/PKBβ, and beta-actin. (D) Boyden chamber trans-well migration assay with U373-DTX1-myc cells not treated (white), treated with a miR-21 inhibitor (α-miR-21, green) or an inhibitor control (α-CTRL , yellow). Average values are shown from at least three individual experiments. Error bars: ±SEM. * p<0.05, *** p<0.001.

To determine this role, we performed transmembrane invasion assays with the two GBM cell lines U373 and LN18 with modified DTX1 expression. DTX1 over-expression did not significantly alter the invasive behavior of U373 cells whereas the U373-shRNA-DTX1 cells showed an average reduction of invasion by 63% compared to control cells (p < 0.001, ***) (Figure 5, A). Cell invasion was elevated in LN18-DTX1 cells to 188% of the EGFP controls (p < 0.001, ***) whereas shRNA-mediated knock-down of DTX1 did not influence the invasive potential of LN18 cells (Figure 5, A). Again we observed a strong effect upon down-regulation of DTX1 in the U373 cell line with high endogenous Notch levels. Similarly, up-regulation of DTX1 in LN18 cells with low endogenous Notch levels increased migration.

To confirm these results in an independent experimental setup, we performed scratch test wound healing assays. Wounds were imaged immediately after the scratch wound was inflicted as well as 12, 24 and 48 hours later. Wound closing, which is a measure for cell motility, was massively reduced in U373-shRNA-DTX1 cells compared to controls (Figure 5, B; Figure S5, A) whereas wound closing was significantly accelerated in LN18-DTX1-myc cells (p < 0.05, *) (Figure 5, B) confirming the initial finding of the transwell migration and invasion assay.

Elevated levels of p-Akt/PKB have been shown to positively correlate with migrating glioma cells, and reduction of total Akt2/PKBβ has been shown to inhibit the migratory as well as the invasive potential of glioma cells [38], [39]. To determine if the alterations in the invasive and migratory potential observed could be explained by these mechanisms, we analyzed the expression pattern of Akt2/PKBβ in our cell lines. We found a positive correlation of Akt2/PKBβ and DTX1 in both cell lines analyzed (Figure 5, C). Akt2/PKBb was down-regulated in shRNA-DTX1 cells to 40% of controls in both cell lines and induced to 120% in U373-DTX-myc and 170% in LN18-DTX1-myc cells (Figure 5, C). The elevated levels of p-Erk in DTX1 over-expressing cells (Figure 2, C) pointed towards a mechanism know to regulate the migratory potential of cells through an Erk/NFκB/Snail1 pathway controlling epithelial to mesenchymal transition (EMT) in primary human mesothelial cells [40]. We therefore analyzed the levels of Snail1 in our glioma cell lines and found a positive correlation with DTX1 (Figure 5, C). Snail1 was down-regulated in shRNA-DTX1 cells to 70% of controls in U373 cells and to 60% in LN18 cells. It was induced to 170% in U373-DTX-myc and 280% in LN18-DTX1-myc cells, which is in accordance to the observed increase in p-Erk levels (Figure 2, C).

Besides targeting a network of tumor suppressor genes, miR-21 has also been described as a migration and invasion promoting factor in gliomas [41]. Therefore we aimed at determining the effect of miR-21 on migration in our DTX1 over-expressing cells. DTX1-myc cells treated with miR-21 inhibitors migrated 19% less than DTX1-myc cells (p < 0.05, *) and 28% less than DTX1-myc cells treated with a control inhibitor (p < 0.001, ***) (Figure 5, D). Akt2/PKBβ induction through DTX1 was neither affected in cells with sip300 nor in cells treated with a miR-21 inhibitor (data not shown). Taken together, we have demonstrated that DTX1 positively correlates with cell migration and invasiveness in U373 and LN18 cells. Furthermore, we have shown that this effect could be mediated by alterations in known pro-migratory signaling pathways in GBM.

### Deltex 1 controls tumor aggressiveness in GBM and breast cancer patients

To elucidate the role of DTX1 in patients, we analyzed the data publicly available at the REMBRANDT database (REpository of Molecular BRAin Neoplasia DaTa, NCI, NIH, USA) to determine if our *in vitro* findings could be brought forward to glioblastoma behavior in patients. GBM patients with intermediate DTX1 expression levels had a median survival time of 402 days. Patients with low levels (reduced by 2 fold or more of intermediate) had a median survival time of 544 days, an increase of more than 35%. Furthermore, the expression of *DTX1*, *PKBβ*, *Snail-1* and *EGR1* correlated with patient survival in GBM patients (Figure S4, A). To assess whether this finding was conferrable to other cancers, we analyzed a data set of early breast cancer samples [42] for the correlation of *DTX1* expression levels and patient survival. Again, prolonged survival in the group with sub-reference *DTX1* expression was detected throughout the period of observation (Figure S4, B). We previously reported that the genetic status of Notch2 has predictive value for GBM survival [10] and patients with high levels of Notch3 have a significantly shorter overall survival (OS) expectancy in the REMBRANDT data set. High levels of Hes1, a marker for canonical Notch pathway activation, correlates with reduced OS as well.

In summary, we found DTX1 expression to be inversely correlated with patient survival in two solid tumor patient cohorts. Notch pathway activation is indicative of a less favorable prognosis. This is in accordance with our *in vitro* data and identifies DTX1 as an oncogenic factor in high grade gliomas.

## Discussion

The functional role of Notch has been intensively studied in human gliomas over the last years. Notch-signaling through distinct receptors regulates critical aspects of glioma biology such as differentiation, proliferation, tumor-stroma interaction and angiogenesis [43]. For example, Notch2 status has been identified as a highly significant prognostic marker in GBM and oligodendroglioma independent of other mutation patterns [10]. Non-canonical Notch ligands have been shown to have tumor inhibiting effects in GBM [20]. However, the role of DTX1, a mediator of non-canonical Notch signaling, has not been elucidated in cancer. DTX1 has been linked to invasiveness in osteosarcoma [44], however, its role in glioma remains elusive. 

In this study we demonstrated an oncogenic role of DTX1 in high grade glioma cell lines. DTX1 increased tumor cell aggressiveness, seen by elevated clonogenicity, increased the migratory and invasive potential of established glioma cells and induced several signaling pathways protecting tumor cells from apoptosis and stimulating survival and proliferation. These effects are linked to a set of genes specifically controlled by DTX1. We will discuss our results in the context of DTX1 as a regulator of gene expression, the regulation of DTX1 itself and of DTX1 as a novel oncogenic factor.

### DTX1 has an oncogenic role in glioma

Oncogenes have the potential to induce malignant growth by conveying uncontrolled proliferation, insensitivity to growth limiting and pro-apoptotic signals, by inducing cell migration, invasion and neo-vascularisation. DTX1 aggravates several of these mechanisms in U373 and LN18 glioma cells. The ability to grow when seeded at low density, as floating spheres or in soft agar indicates abnormal growth potential and increased resistance to anoikis. These phenotypes are enhanced by high DTX1 levels. Furthermore, GBM patients with low *DTX1* expression levels have a better prognosis and have a >35% longer median survival than controls. The increased levels of RTK/PI3K/PKB and MAPK/ERK activation observed in DTX1-myc cells offer a possible mechanistic explanation for this enhanced tumorigenic potential observed in patients with normal DTX1 levels. The elevated protein levels of the anti-apoptotic factor Mcl-1 point to a second mode of action. Interestingly, a link between Notch receptors and Mcl-1 had previously been demonstrated [45]. However, this effect could not be assigned to the canonical factors MAML1 and RBPJκ. Taken together, these findings suggest that Mcl-1 is specifically controlled through the non-canonical Notch pathway. DTX1 mediated effects may be conferrable to other solid tumors. For example, the better prognosis of early breast cancer patients with low *DTX1* expression levels points in this direction.

Massive tumor cell migration and brain invasion are hallmarks of GBM. These migrating cells pose a major obstacle to any successful therapy, thwarting complete surgical resection. DTX1 positively correlates with the migratory and invasive potential of U373 and LN18 cells. This effect correlates with the levels of endogenous Notch within the cancer cell. For example, in U373 cells, which have high levels of endogenous Notch, DTX1 only alters the migratory behavior upon down-regulation, suggesting a saturated activation of the Notch/DTX pathway. In low Notch expressing LN18 cells the opposite is observed; down-regulating DTX1 shows little effect indicating inactive Notch/DTX signaling whereas DTX1 over-expression induces massive changes. Although the pro-migratory factors Snail-1 and Akt2/PKBβ are influenced by DTX1 in both lines, high Notch activity appears to saturate the migratory behavior and cannot be further induced by over-expressing DTX1 whereas low Notch levels readily induce migration. The MAPK/ERK pathway has previously been linked to proliferation and migration in glioblastoma [46]. DTX1 levels strongly correlate with the levels of p-Erk in all cell lines analyzed independent of Notch levels. Furthermore, MMP-9, an endopeptidase that digests basement membrane type IV collagen, is induced by p-Erk in glioma cells [47]. Therefore, elevated levels of p-Erk could explain the increased invasive potential while the elevated levels of Akt2/PKBβ act on cell adhesion and cytoskeleton rearrangement [39] leaving the cells with significantly aggravated invasive and migratory behavior as observed in our experiments.

### DTX1 controls the expression of oncogenes, tumor suppressors and oncomirs in glioma

Deltex has been shown to be a negative regulator of Notch signaling in cancer cells by targeting the Notch-ICD for poly-ubiquitination and degradation [44]. Notch signaling itself induces Deltex which thereby leads to a negative feedback loop. The high degree of similarity in our overall gene expression profiles between MAML1-dn and DTX1-myc cells further supports this model. However, DTX1 also controls genes which are not regulated by the canonical Notch pathway and have a direct impact on cancer progression, e.g. miR-21 which has a well-established role as an oncomir through several modes of action [36], [48], [49]. Our results demonstrate miR-21 activation through non-canonical Notch signaling to depend on p300 levels. In *C. elegans* LIN-12/Notch signaling has previously been linked to microRNA expression [50]. The two Notch signaling pathways may represent an evolutionary conserved mechanism of microRNA regulation.

DUSP6, a gene down regulated in our DTX1 over-expressing cells, belongs to a class of dual-specificity phosphatases which dephosphorylate MAPK pathway proteins ERK, JNK, and p38 [51]. This offers a direct functional link between DTX1 specific gene expression and p-Erk levels. Additionally, we found APPL1, a co-activator of Akt2/PKBβ [52], to be up-regulated by DTX1. Both have a similar activating effect on cell migration in glioma cells. It is not clear to which extent the change in the invasive potential of the cells is mediated by these pathways or by miR-21, which also has been described as a regulator of invasiveness. However, inhibition of miR-21 alone did not reduce migration as much as down regulation of DTX1 did, indicating an additive, combinatorial effect of several mechanisms controlled by DTX1.

Altogether, DTX1 activates a set of transcripts with oncogenic functions and down regulates tumor suppressors in parallel. How these different aspects interact and to which extent the changes in expression are direct or indirect remain to be established. An additional layer of complexity is added by the fact that not all changes in gene expression or signaling are p300 dependent in our experiments. Whereas miR-21, Snail-1 or Mcl-1 expression was reduced in cells with diminished p300 levels, this was not the case for Akt2/PKBβ or Erk. Several modes of action could explain this finding. DTX1 has been shown to bind to p300 and thereby sequester it from its previous binding partners. This effect leads to the inhibition of the transcription factor MASH1 which controls differentiation in neural progenitor cells [24]. If the change induced by DTX1 is caused by reducing p300 availability at a certain site, a further reduction of p300 by siRNA would not further enhance the initial alteration. Non-canonical Notch signaling also inhibits E47, a widely expressed transcription factor [53]. Therefore, DTX1 could regulate gene expression directly by forming a DNA binding complex with p300 and thereby reducing its availability and/or by changing the activity of other transcription factor complexes. DTX1 has been shown to be an E3-Ub-ligase tagging proteins for proteasomal degradation. To which extent the observed phenotypes depend on this function cannot be answered from our data.

### Deltex can be activated by Notch and other signaling cascades

If the DTX1 specific transcriptional program is activated by specific ligands of Notch receptors as it was shown for oligodendrocyte maturation, which is induced by F3/Contactin [17], remains to be determined. The NB-3/Notch1 pathway also has been reported to signal specifically through DTX1 but not through the canonical Notch pathway [54]. Different DTX1 specific ligands appear to have very distinct effects as DNER has been reported to reduce the tumorigenic potential of GBM derived cells [20]. However, it is unclear if DTX1 was induced in these cells or not.

Although Notch receptors are the best established activators of Deltex, there are also other mechanisms described. The transcription factor NFAT (nuclear factor of activated T cells) was shown to induce DTX1 expression in T cells where DTX1 then acted as a transcriptional regulator independently of Notch [55]. Granule cell precursors of the cerebellum increase *DTX1* expression when stimulated with Shh [56]. If this induction of Deltex occurred directly or if this was a secondary effect due to overall changes in gene expression remained unaddressed. Regarding GBM, DTX1 is induced by Notch signaling, however, alternative pathways like SHH may also contribute to direct activation of DTX1. It remains to be shown in glioma if NB3, F3/contactin and other specific ligands to Notch receptors exclusively signal through the non-canonical pathway. In conclusion, we propose the alternative Notch pathway via DTX1 as an oncogenic factor in malignant glioma. We found low *DTX1* expression levels to correlate with prolonged survival in solid tumor patients.

## Supporting Information

Figure S1 **Heat map of differential gene expression in glioma cell lines with modified Notch canonical and non-canonical signaling.** (A) Western blot analysis of Notch1/2 expression in DTX1 over-expressing U373 cells probing for Notch1, Notch2, Myc-tag (DTX1) and actin. (B) Protein blots showing MAML1-dn expression in U373 cells used for microarray analysis. Numbers in (A) and (B): relative densitometry values of blots. (C) Heat map: gene expression values are shown as color coded heat map with red representing low and white representing high expression values. The three samples are listed on the x-axis, individual genes on the y-axis. Gene clustering was performed according to similarity in expression pattern. All genes shown are differentially expressed in at least one sample (fold change of expression >2, p-value < 0.01). Asterisks indicate example areas with unique expression patterns in DTX1-myc cells. (D) Venn-Diagram of gene expression analysis. Genes differentially expressed in U373-DTX1-myc are shown in red, genes differentially expressed in U373-MAML1-dn are shown in blue, genes altered in both cell lines are shown in purple.
(TIF)

Figure S2 **Dendrograms based on gene expression profiles of glioma cell lines.** Dendrograms based on the gene expression profiles of the glioma cell lines used visualizing relatedness of samples based on (A) overall gene expression pattern including all 22’000 genes annotated on the microarray and (B) based on the 191 genes indentified to be specifically controlled by DTX1. *p*-values were calculated via multiscale bootstrap resampling.
(TIF)

Figure S3 **Colony formation and cell density are changed by DTX1 expression modification.** (A) Low density cell seeding of glioma cells showing positive correlation between colony formation potential and DTX1 levels in glioma cell lines U373 and LN18. (B) Low density seeding colonies shown as light microscopic pictures demonstrating the aggregation phenotype in LN18-DTX1-myc cells. Arrows point to the center of individual colonies indicating the area of aggregation in LN18-DTX1-myc cells. Close up images show borders of colonies at higher magnification. (C) Low density seeding of U373-DTX1-myc cells treated with miR-21 inhibitor (α-miR-21) or control inhibitor (α-CTRL). (D) Quantification of low density seeding shown in (C). Average values are shown. Error bars: ±SEM.
(TIF)

Figure S4 **Survival curves of GBM and early breast cancer patients.** (A) Survival data derived from the REMBRANDT database for selected genes of interest: *DTX1, PKBβ, SNAI1, EGR1*. (B) Kaplan-Meier survival plot of early breast cancer patients with sub-reference *DTX1* expression (blue) compared to above-reference *DTX1* expression levels (red).
(TIF)

Figure S5 **Light-microscopic images of wound healing assay.** (A) Light-microscopic images of GBM cells in the 48h wound closing assay. U373-shRNA-scr control and U373-shRNA-DTX1 cells were imaged immediately after the wound was inflicted (0h), after one day (24h), and after two days (48h). Dashed lines indicate approximate line of wound edges.
(TIF)

Table S1 **Summary of gene expression changes.**
(DOCX)

Table S2 **Gene onthology analysis of differentially expressed genes.**
(DOCX)

Table S3 **Complete list of differential expressed genes.**
(DOCX)
