# Supplementary figures and images for: Deltex-1 Activates Mitotic Signaling and Proliferation and Increases the Clonogenic and Invasive Potential of U373 and LN18 Glioblastoma Cells and Correlates with Patient Survival
Source: PLoS One. 2013 Feb 25;8(2):e57793. doi: 10.1371/journal.pone.0057793 (PMC3581491; doi:10.1371/journal.pone.0057793)

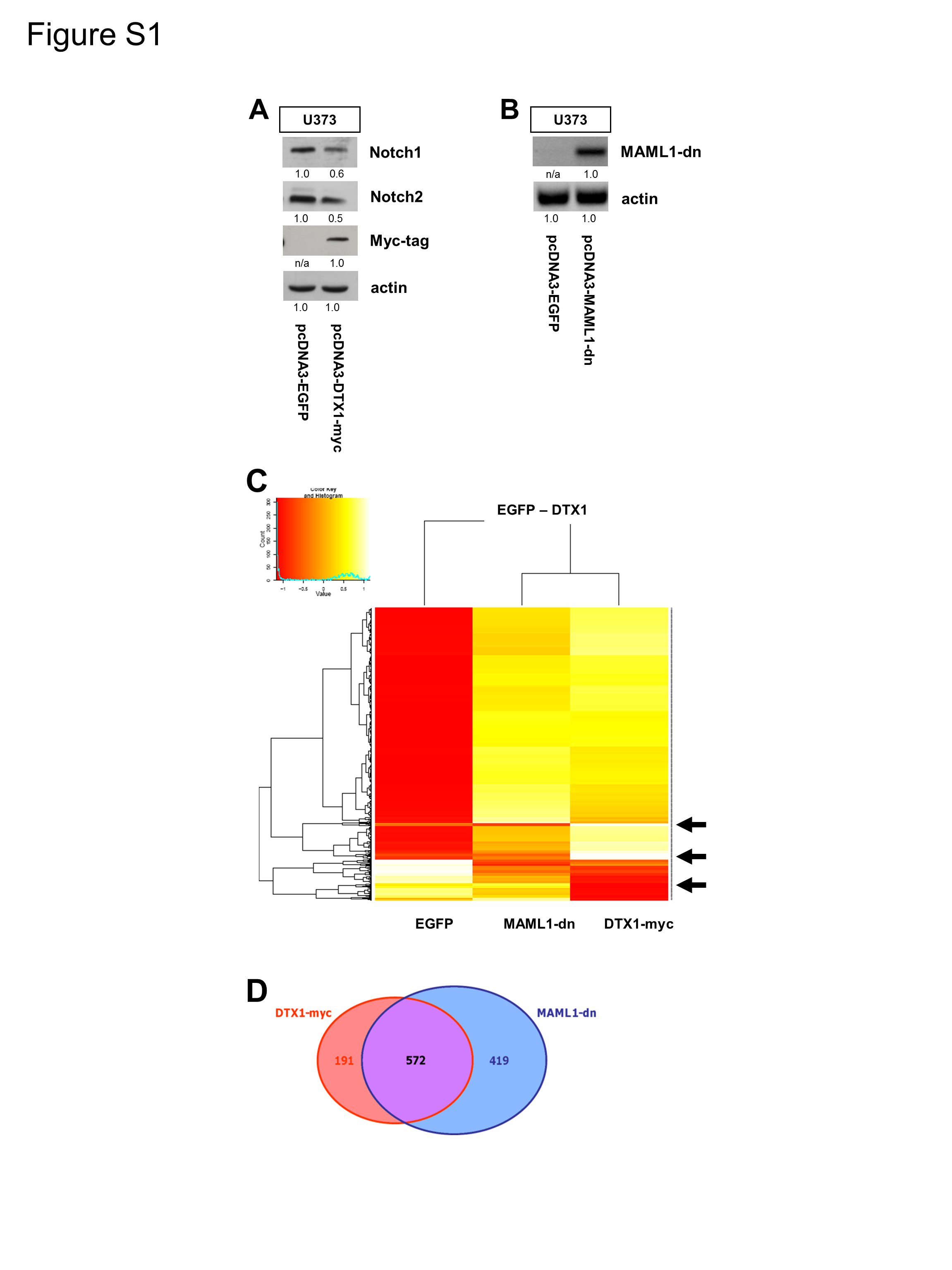

Supplement: Figure S1 — Heat map of differential gene expression in glioma cell lines with modified Notch canonical and non-canonical signaling. (A) Western blot analysis of Notch1/2 expression in DTX1 over-expressing U373 cells probing for Notch1, Notch2, Myc-tag (DTX1) and actin. (B) Protein blots showing MAML1-dn expression in U373 cells used for microarray analysis. Numbers in (A) and (B): relative densitometry values of blots. (C) Heat map: gene expression values are shown as color coded heat map with red representing low and white representing high expression values. The three samples are listed on the x-axis, individual genes on the y-axis. Gene clustering was performed according to similarity in expression pattern. All genes shown are differentially expressed in at least one sample (fold change of expression >2, p-value < 0.01). Asterisks indicate example areas with unique expression patterns in DTX1-myc cells. (D) Venn-Diagram of gene expression analysis. Genes differentially expressed in U373-DTX1-myc are shown in red, genes differentially expressed in U373-MAML1-dn are shown in blue, genes altered in both cell lines are shown in purple. (TIF) [file pone.0057793.s001.tif]

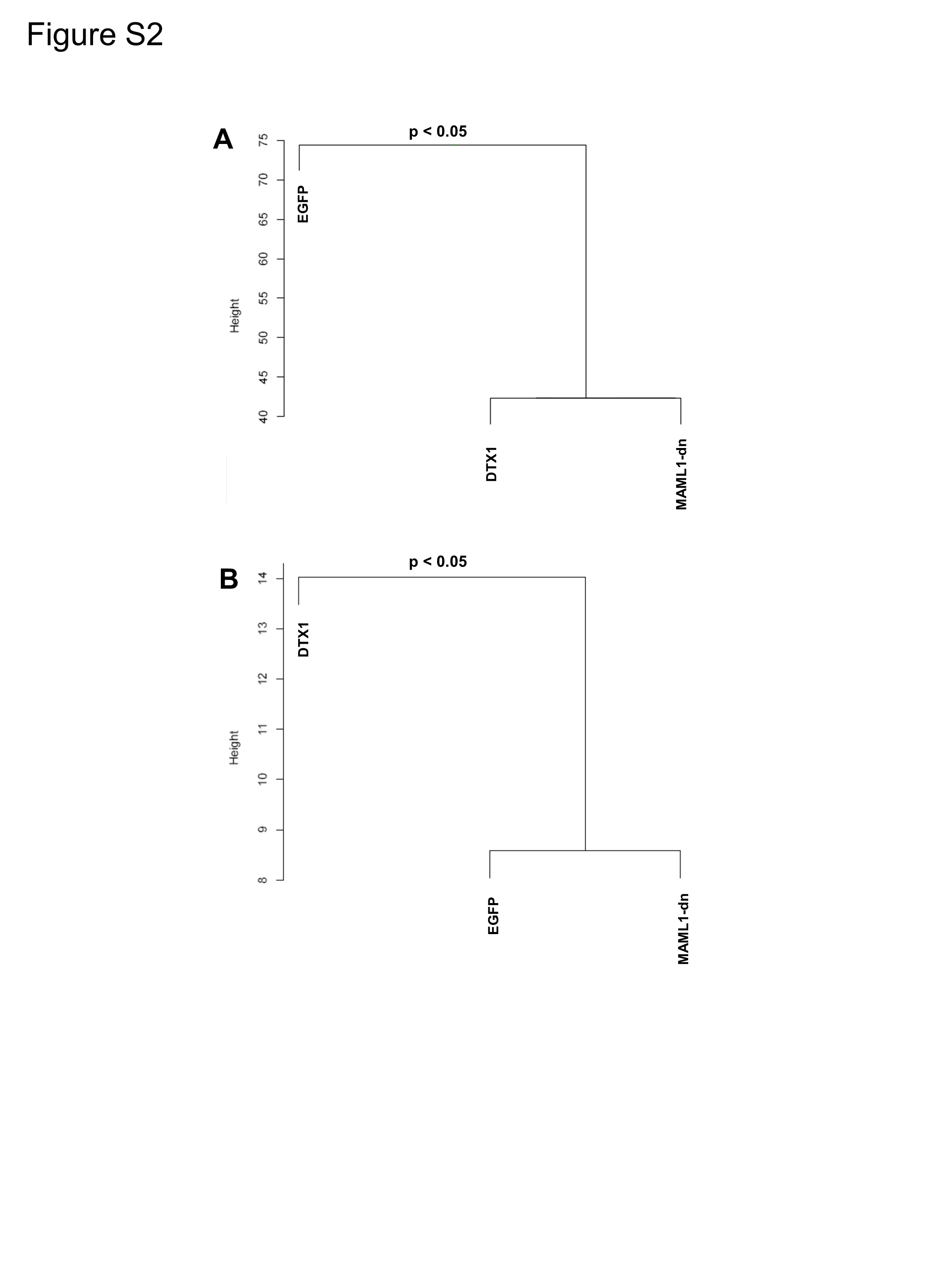

Supplement: Figure S2 — Dendrograms based on gene expression profiles of glioma cell lines. Dendrograms based on the gene expression profiles of the glioma cell lines used visualizing relatedness of samples based on (A) overall gene expression pattern including all 22’000 genes annotated on the microarray and (B) based on the 191 genes indentified to be specifically controlled by DTX1. p-values were calculated via multiscale bootstrap resampling. (TIF) [file pone.0057793.s002.tif]

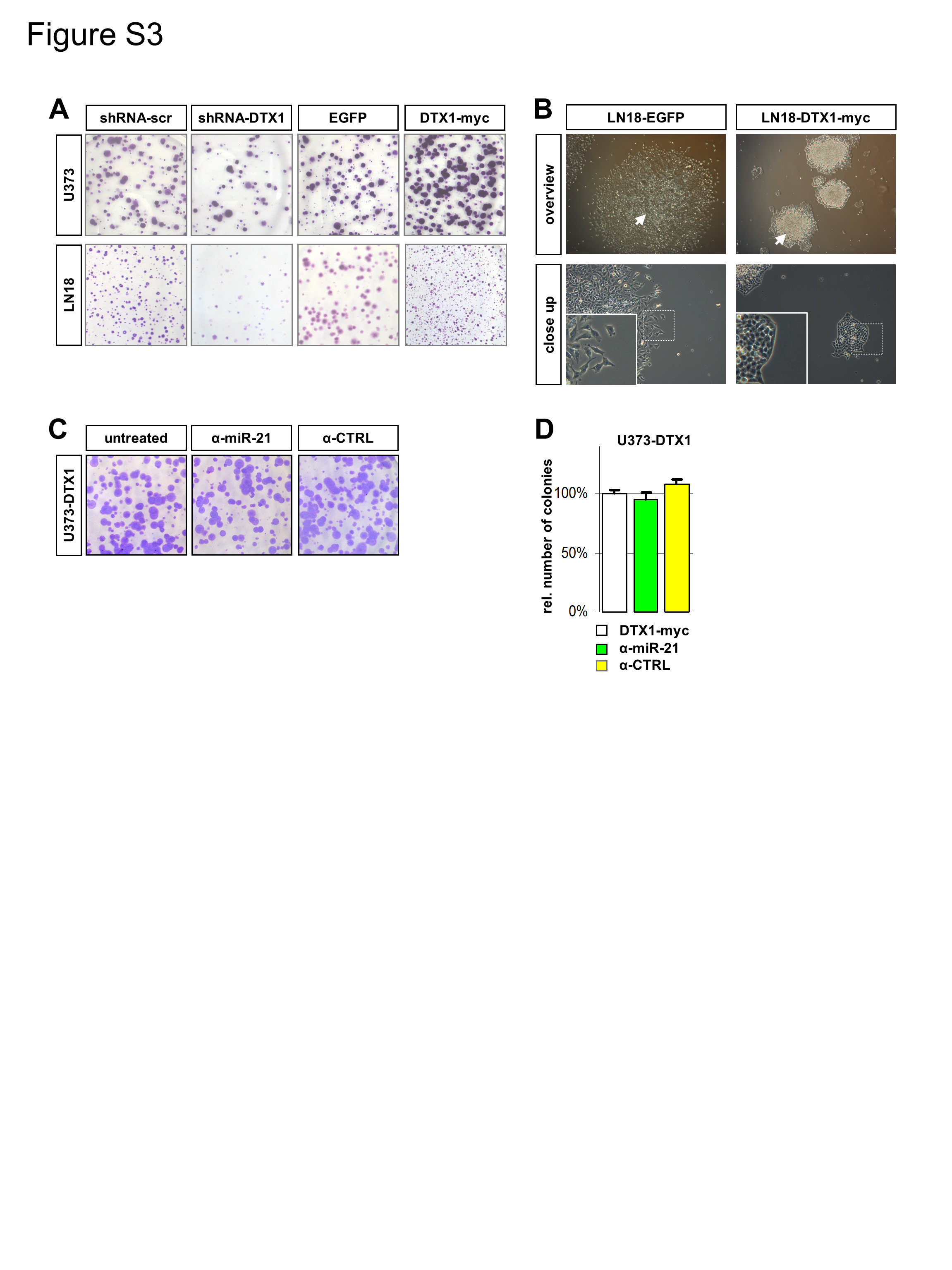

Supplement: Figure S3 — Colony formation and cell density are changed by DTX1 expression modification. (A) Low density cell seeding of glioma cells showing positive correlation between colony formation potential and DTX1 levels in glioma cell lines U373 and LN18. (B) Low density seeding colonies shown as light microscopic pictures demonstrating the aggregation phenotype in LN18-DTX1-myc cells. Arrows point to the center of individual colonies indicating the area of aggregation in LN18-DTX1-myc cells. Close up images show borders of colonies at higher magnification. (C) Low density seeding of U373-DTX1-myc cells treated with miR-21 inhibitor (α-miR-21) or control inhibitor (α-CTRL). (D) Quantification of low density seeding shown in (C). Average values are shown. Error bars: ±SEM. (TIF) [file pone.0057793.s003.tif]

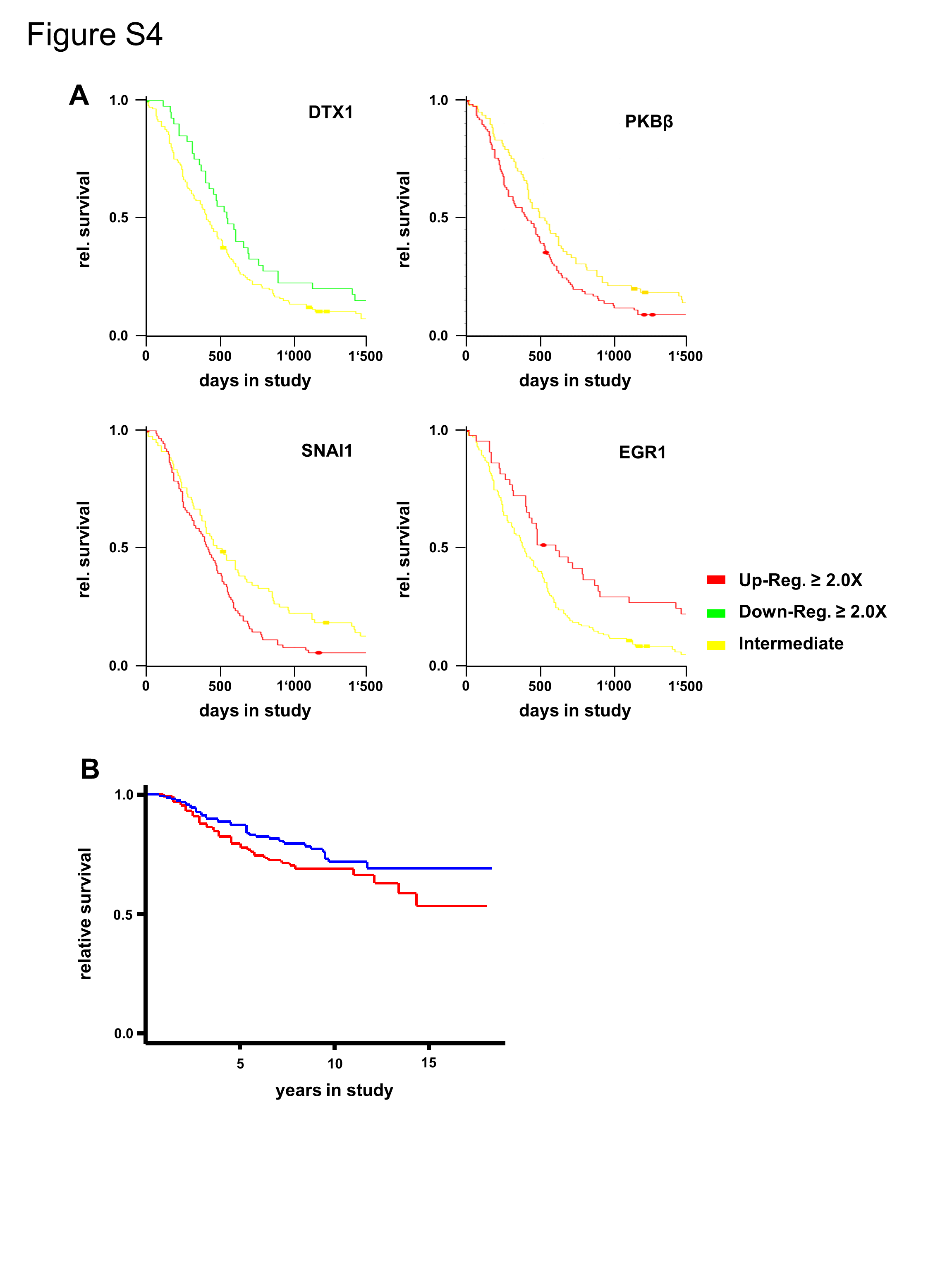

Supplement: Figure S4 — Survival curves of GBM and early breast cancer patients. (A) Survival data derived from the REMBRANDT database for selected genes of interest: DTX1, PKBβ, SNAI1, EGR1. (B) Kaplan-Meier survival plot of early breast cancer patients with sub-reference DTX1 expression (blue) compared to above-reference DTX1 expression levels (red). (TIF) [file pone.0057793.s004.tif]
