# Supplementary material for: Deltex-1 Activates Mitotic Signaling and Proliferation and Increases the Clonogenic and Invasive Potential of U373 and LN18 Glioblastoma Cells and Correlates with Patient Survival
Source: PLoS One. 2013 Feb 25;8(2):e57793. doi: 10.1371/journal.pone.0057793 (PMC3581491; doi:10.1371/journal.pone.0057793)
Supplement: Table S1 — Summary of gene expression changes. (DOCX) [file pone.0057793.s006.docx]

**Table S1, summary of gene expression changes**

| **number of genes changed in DTX1-myc cells** | |
| --- | --- |
| up regulated in DTX1 vs. EGFP | 665 |
| down regulated in DTX1 vs. EGFP | 98 |
|  |  |
| **number of genes changed in MAML1-dn cells** | |
| up regulated in MAML1-dn vs. EGFP | 872 |
| down regulated in MAML1-dn vs. EGFP | 119 |
|  |  |
| **number of genes commonly changed in both cell lines** | |
| up regulated in DTX1 and MAML1-dn vs. EGFP | 542 |
| down regulated in DTX1 and MAML1-dn vs. EGFP | 30 |
|  |  |
| **number of genes uniquely changed in DTX1-myc cells** | |
| up regulated in DTX1 vs. EGFP and MAML1-dn | 121 |
| down regulated in DTX1 vs. EGFP and MAML1-dn | 70 |
