# Supplementary material for: Deltex-1 Activates Mitotic Signaling and Proliferation and Increases the Clonogenic and Invasive Potential of U373 and LN18 Glioblastoma Cells and Correlates with Patient Survival
Source: PLoS One. 2013 Feb 25;8(2):e57793. doi: 10.1371/journal.pone.0057793 (PMC3581491; doi:10.1371/journal.pone.0057793)
Supplement: Table S2 — Gene onthology analysis of differentially expressed genes. (DOCX) [file pone.0057793.s007.docx]

**Table S2, gene onthology analysis of differentially expressed genes**

| **Number of genes per gene onthology class** | | |
| --- | --- | --- |
| metabolic process | 23 | 12% |
| signal transduction | 22 | 12% |
| cell cycle | 15 | 8% |
| transcription | 14 | 7% |
| intracellular transport | 14 | 7% |
| protein modification process | 13 | 7% |
| cell motion | 6 | 3% |
| cell adhesion / extra cellular matrix | 5 | 3% |
| cell homeostasis | 5 | 3% |
| apoptosis | 4 | 2% |
| immune response | 3 | 2% |
| translation | 3 | 2% |
|  |  |  |
| others | 41 | 21% |
| n/a | 23 | 12% |
