# Supplementary material for: Deltex-1 Activates Mitotic Signaling and Proliferation and Increases the Clonogenic and Invasive Potential of U373 and LN18 Glioblastoma Cells and Correlates with Patient Survival
Source: PLoS One. 2013 Feb 25;8(2):e57793. doi: 10.1371/journal.pone.0057793 (PMC3581491; doi:10.1371/journal.pone.0057793)
Supplement: Table S3 — Complete list of differential expressed genes. (DOCX) [file pone.0057793.s008.docx]

**Table S3, complete list of differential expressed genes**

| **Accession ID** | **Gene Symbol** | **log FC** |
| --- | --- | --- |
| NM_004416 | DTX1 | 5.56 |
| NM_001305 | CLDN4 | 4.49 |
| NM_006988 | ADAMTS1 | 2.16 |
| NM_016542 | RP6-213H19.1 | 2.02 |
| NM_000465 | BARD1 | 1.91 |
| NM_000047 | ARSE | 1.8 |
| NM_018165 | PBRM1 | 1.68 |
| NM_153020 | RBM24 | 1.62 |
| NM_000339 | SLC12A3 | 1.62 |
| NM_005264 | GFRA1 | 1.58 |
| NM_001992 | F2R | 1.57 |
| NM_001786 | CDC2 | 1.56 |
| NM_024639 | ZNF322A | 1.54 |
| NM_006625 | FUSIP1 | 1.54 |
| NM_016816 | OAS1 | 1.54 |
| NM_004523 | KIF11 | 1.53 |
| NM_018303 | EXOC2 | 1.53 |
| NM_000138 | FBN1 | 1.52 |
| AY699265 | MIRN21 | 1.51 |
| NM_198098 | AQP1 | 1.51 |
| NM_019054 | FAM35A | 1.49 |
| NM_012334 | MYO10 | 1.44 |
| BC010491 | C2orf59 | 1.43 |
| NM_007112 | THBS3 | 1.41 |
| NM_007027 | TOPBP1 | 1.41 |
| NM_013352 | DSE | 1.39 |
| NM_006317 | BASP1 | 1.38 |
| NM_053276 | VIT | 1.37 |
| NM_001018159 | NAE1 | 1.35 |
| NM_015446 | AHCTF1 | 1.34 |
| NM_015255 | UBR2 | 1.33 |
| NM_014333 | CADM1 | 1.32 |
| NM_014363 | SACS | 1.32 |
| NM_018131 | CEP55 | 1.31 |
| NM_007296 | BRCA1 | 1.31 |
| NM_002806 | PSMC6 | 1.31 |
| NM_005124 | NUP153 | 1.3 |
| NM_022113 | KIF13A | 1.29 |
| NM_018243 | Sep 11 | 1.27 |
| NM_005559 | LAMA1 | 1.26 |
| NM_014783 | ARHGAP11A | 1.26 |
| NM_003358 | UGCG | 1.25 |
| NM_015382 | HECTD1 | 1.25 |
| NM_015295 | SMCHD1 | 1.25 |
| NM_012096 | APPL1 | 1.24 |
| NM_024776 | SGK269 | 1.24 |
| NM_133265 | AMOT | 1.23 |
| NM_032485 | MCM8 | 1.23 |
| NM_003107 | SOX4 | 1.22 |
| NM_002806 | PSMC6 | 1.22 |
| NM_014969 | WDR47 | 1.22 |
| NM_005385 | NKTR | 1.2 |
| NM_001204 | BMPR2 | 1.19 |
| NM_080927 | DCBLD2 | 1.19 |
| NM_007235 | XPOT | 1.18 |
| NM_012124 | CHORDC1 | 1.18 |
| NM_006638 | RPP40 | 1.18 |
| NM_001797 | CDH11 | 1.17 |
| NM_001128205 | SULF1 | 1.17 |
| NM_005160 | ADRBK2 | 1.17 |
| NM_016513 | ICK | 1.16 |
| NM_020319 | ANKMY2 | 1.16 |
| NM_001821 | CHML | 1.16 |
| NM_022841 | RFX7 | 1.16 |
| NM_019054 | FAM35A | 1.16 |
| NM_138771 | CCDC126 | 1.15 |
| NM_015199 | ANKRD28 | 1.15 |
| NM_015446 | AHCTF1 | 1.15 |
| NM_004162 | RAB5A | 1.15 |
| NM_017437 | CPSF2 | 1.14 |
| NM_001042517 | DIAPH3 | 1.14 |
| NM_015384 | NIPBL | 1.13 |
| NM_001624 | AIM1 | 1.12 |
| NM_005246 | FER | 1.12 |
| NM_007159 | SLMAP | 1.11 |
| NM_003238 | TGFB2 | 1.11 |
| NM_006708 | GLO1 | 1.11 |
| NM_178862 | STT3B | 1.1 |
| NM_001025366 | VEGFA | 1.1 |
| NM_013296 | GPSM2 | 1.09 |
| NM_002759 | EIF2AK2 | 1.09 |
| NM_003341 | UBE2E1 | 1.08 |
| NM_080546 | SLC44A1 | 1.08 |
| NM_015092 | SMG1 | 1.08 |
| NM_003368 | USP1 | 1.08 |
| NM_022459 | XPO4 | 1.07 |
| NM_003798 | CTNNAL1 | 1.06 |
| NM_199132 | ZNF468 | 1.06 |
| NM_005746 | NAMPT | 1.06 |
| NM_003162 | STRN | 1.06 |
| NM_003659 | AGPS | 1.05 |
| NM_024769 | ASAM | 1.05 |
| AK295862 | KIAA0528 | 1.05 |
| NM_021038 | MBNL1 | 1.04 |
| NM_007085 | FSTL1 | 1.04 |
| NM_012158 | FBXL3 | 1.04 |
| NM_001123390 | TBC1D3H | 1.04 |
| NM_005921 | MAP3K1 | 1.04 |
| NM_019024 | HEATR5B | 1.04 |
| NM_012253 | TKTL1 | 1.04 |
| NM_018010 | IFT57 | 1.03 |
| NM_006101 | NDC80 | 1.03 |
| NM_020165 | RAD18 | 1.03 |
| NM_020923 | ZDBF2 | 1.03 |
| ENST00000321331 | HIGD1A | 1.03 |
| NM_015054 | UHRF1BP1L | 1.03 |
| NM_153362 | PRSS35 | 1.02 |
| NM_000213 | ITGB4 | 1.02 |
| NM_006355 | TRIM38 | 1.02 |
| AK094159 | FLJ36840 | 1.02 |
| NM_015097 | CLASP2 | 1.02 |
| NM_206855 | QKI | 1.01 |
| ENST00000379607 | EIF1AX | 1.01 |
| NM_001113546 | LIMA1 | 1.01 |
| NM_175866 | UHMK1 | 1.01 |
| ENST00000308482 | LRRFIP1 | 1.01 |
| NM_017801 | CMTM6 | 1.01 |
| NM_001024457 | RGPD1 | 1.01 |
| NM_001257 | CDH13 | 1.01 |
| NM_020748 | INTS2 | 1 |
| NM_174942 | GAS2L3 | 1 |
|  |  |  |
| NM_000693 | ALDH1A3 | -1 |
| NM_000014 | A2M | -1.01 |
| BC043250 | LOC732275 | -1.02 |
| NM_016234 | ACSL5 | -1.02 |
| NM_001114618 | MGAT1 | -1.04 |
| NM_014365 | HSPB8 | -1.04 |
| NM_005252 | FOS | -1.04 |
| NM_001772 | CD33 | -1.05 |
| NM_033549 | TRIM41 | -1.05 |
| NM_012410 | SEZ6L2 | -1.05 |
| NM_001964 | EGR1 | -1.06 |
| NM_001217 | CA11 | -1.06 |
| NM_003009 | SEPW1 | -1.07 |
| NM_022170 | EIF4H | -1.08 |
| NM_006598 | SLC12A7 | -1.08 |
| NM_000155 | GALT | -1.09 |
| NM_030964 | SPRY4 | -1.1 |
| NM_004041 | ARRB1 | -1.1 |
| NM_018192 | LEPREL1 | -1.11 |
| NM_000358 | TGFBI | -1.12 |
| NM_012306 | FAIM2 | -1.12 |
| NM_002231 | CD82 | -1.13 |
| AK302783 | LOC728212 | -1.14 |
| NM_024615 | PARP8 | -1.14 |
| NM_018044 | NSUN5 | -1.15 |
| NM_016086 | STYXL1 | -1.15 |
| NM_032564 | DGAT2 | -1.17 |
| NM_006435 | IFITM2 | -1.17 |
| NM_004283 | RAB3D | -1.17 |
| NM_198282 | TMEM173 | -1.18 |
| NM_018110 | DOK4 | -1.18 |
| NM_020645 | NRIP3 | -1.22 |
| NM_002607 | PDGFA | -1.23 |
| NM_173619 | MGC34761 | -1.23 |
| NM_145662 | SPANXA2 | -1.26 |
| NM_001036 | RYR3 | -1.26 |
| NM_015253 | WSCD1 | -1.27 |
| NM_001946 | DUSP6 | -1.28 |
| NM_005582 | CD180 | -1.3 |
| NM_000170 | GLDC | -1.3 |
| NM_001164 | APBB1 | -1.32 |
| BC033537 | KIAA1576 | -1.34 |
| NM_001020818 | MYADM | -1.35 |
| NM_024022 | TMPRSS3 | -1.37 |
| NM_003283 | TNNT1 | -1.38 |
| NM_021939 | FKBP10 | -1.38 |
| NM_002065 | GLUL | -1.39 |
| NM_173357 | SSX6 | -1.45 |
| NM_020826 | SYT13 | -1.45 |
| AK058065 | LOC100128840 | -1.5 |
| NM_001089 | ABCA3 | -1.51 |
| NM_000852 | GSTP1 | -1.51 |
| AF503509 | C1orf32 | -1.52 |
| NM_052896 | CSMD2 | -1.52 |
| NM_004530 | MMP2 | -1.54 |
| NM_005330 | HBE1 | -1.58 |
| NM_002606 | PDE9A | -1.6 |
| NM_014244 | ADAMTS2 | -1.7 |
| NM_002346 | LY6E | -1.72 |
| NM_020422 | TMEM159 | -1.72 |
| NM_003811 | TNFSF9 | -1.82 |
| NR_002223 | TPRXL | -1.84 |
| NM_001040058 | SPP1 | -1.9 |
| NM_001354 | AKR1C2 | -2.05 |
| AY513283 | GLUL | -2.12 |
| NM_012188 | FOXI1 | -2.39 |
| NM_001958 | EEF1A2 | -2.48 |
| NM_003835 | RGS9 | -2.65 |
| NM_001175 | ARHGDIB | -2.75 |
| NM_001025195 | CES1 | -3.06 |
